# Novel *ATP7A* Splice-Site Variant Causing Distal Motor Neuropathy and Occipital Horn Syndrome: Two Siblings and Literature Review

**DOI:** 10.3390/genes16091077

**Published:** 2025-09-15

**Authors:** Karin Writzl, Maruša Škrjanec Pušenjak, Matevž Jus, Aleš Maver, Nuška Pečarič Meglič, Borut Peterlin, Lea Leonardis

**Affiliations:** 1 Clinical Institute of Genomic Medicine, University Medical Centre Ljubljana, 1000 Ljubljana, Slovenia; karin.writzl@kclj.si (K.W.); marusa.skrjanec@kclj.si (M.Š.P.); matevz.jus@ukc-mb.si (M.J.); ales.maver@kclj.si (A.M.); borut.peterlin@kclj.si (B.P.); 2 Medical Faculty, University Ljubljana, 1000 Ljubljana, Slovenia; 3 Clinical Institute of Genetic Diagnostics, University Medical Centre Maribor, 2000 Maribor, Slovenia; 4 Clinical Institute of Radiology, University Medical Centre Ljubljana, 1000 Ljubljana, Slovenia; nuska.pecaric@kclj.si; 5 Institute of Clinical Neurophysiology, Division of Neurology, University Medical Center Ljubljana, 1525 Ljubljana, Slovenia; 6 Department of Neurology, Faculty of Medicine, University of Ljubljana, 1000 Ljubljana, Slovenia

**Keywords:** *ATP7A*, splice-site variant, distal hereditary motor neuropathy, occipital horn syndrome, copper metabolism, neurogenetics

## Abstract

**Background:** Pathogenic hemizygous variants in *ATP7A* most commonly cause Menkes disease or occipital horn syndrome (OHS), whereas *ATP7A*-related distal hereditary motor neuropathy (dHMN) is rarely reported. Here, we describe two adult brothers with an overlapping dHMN/OHS phenotype caused by a novel *ATP7A* splice-site variant and review the clinical and genetic features of previously published patients with *ATP7A*-related dHMN. **Methods:** We performed detailed clinical, electrophysiological, and genetic evaluations of both siblings, including exome sequencing and RNA analysis. Additionally, we reviewed the clinical, electrophysiological, and genetic data of previously reported patients with *ATP7A*-related dHMN. **Results:** We identified a novel hemizygous *ATP7A* splice-site variant (NM_000052.7:c.1544-2A>T) in both brothers. The younger brother, who exhibited a more severe phenotype, presented in early childhood with mild global developmental delay, intellectual disability, and chronic diarrhea, while the older brother had childhood-onset chronic diarrhea without cognitive impairment. Both developed distal hereditary motor neuropathy later in life, and imaging revealed occipital horns. Serum copper and ceruloplasmin levels were mildly reduced. RNA sequencing revealed two aberrant transcript isoforms resulting from the splice-site variant, one of which may produce a partially functional protein. Review of previously reported patients shows that *ATP7A*-related dHMN may occur isolated or with overlapping features of OHS. In patients with the overlapping phenotype, chronic diarrhea was often the first symptom, followed by slowly progressive dHMN. **Conclusions:** Previously reported *ATP7A*-related dHMN has been mostly associated with missense variants. Our findings expand the mutational spectrum by identifying a splice-site variant. In patients with an overlapping OHS/dHMN phenotype, diagnosis was typically delayed for decades, suggesting this presentation remains underdiagnosed.

## 1. Introduction

Pathogenic hemizygous variants in the gene encoding the copper transporter ATP7A cause an X-linked recessive spectrum of disorders with variable clinical presentations, including classic Menkes disease (MD, MIM #309400), occipital horn syndrome (OHS, MIM #304150), and *ATP7A*-related distal hereditary motor neuropathy (dHMN, MIM #300489) [1].

*ATP7A* encodes a P-type ATPase copper transporter that plays a central role in copper homeostasis [2]. Under basal copper conditions, ATP7A localizes to the trans-Golgi network (TGN), where it supplies copper to copper-dependent enzymes, such as cytochrome c oxidase, superoxide dismutase, lysyl oxidase, and others. When intracellular Cu concentrations rise, ATP7A redistributes from the TGN to post-Golgi compartments and recycling endosomes to mediate copper export. Loss of ATP7A activity therefore results in systemic copper deficiency and impaired function of copper-dependent enzymes, particularly affecting the brain and connective tissue [3].

The clinical spectrum of *ATP7A*-related disorders is broad. MD and OHS are considered part of the same disease spectrum. MD represents the most severe end of the clinical spectrum, characterized by progressive infantile-onset, severe cerebral and cerebellar neurodegeneration and connective tissue abnormalities. At the milder end is OHS, presenting with absent or mild neurodevelopment abnormalities, bony exostosis (occipital horns), dysautonomia, and connective tissue anomalies. In both MD and OHS, serum copper and ceruloplasmin concentrations are typically reduced. Intermediate MD-OHS phenotypes share clinical and biochemical features of both diseases [4,5,6,7]. In contrast to MD and OHS, dHMN is an adult-onset disorder resembling Charcot–Marie–Tooth disease, which has been reported in only a small number of patients. It was initially described as an isolated clinical presentation, not associated with any features of MD or OHS, and with normal serum copper and ceruloplasmin concentrations [8,9]. However, more recently, four patients with dHMN in combination with dysautonomia and connective tissue abnormalities have been reported, suggesting an overlapping phenotype between dHMN and OHS [10,11,12].

While in MD and OHS disease, severity largely correlates with the degree of residual ATP7A activity, dHMN follows a distinct mechanism not explained by loss-of-function alone. Loss-of-function variants, such as nonsense variants and large deletions, where the function of ATP7A is entirely or almost entirely lost, cause MD [7,13]. Variants that permit partial protein activity, often splice-site variants, are associated with OHS [7,13]. In contrast, missense variants clustered in the C-terminal region of ATP7A cause dHMN, an adult-onset motor neuron disorder typically associated with normal or only mildly reduced serum copper and ceruloplasmin concentrations [8,9]. Functional studies indicate that these variants impair ATP7A trafficking and copper homeostasis in neurons [8,9].

We report two adult brothers with an overlapping phenotype of dHMN and OHS with a novel splice-site variant in *ATP7A*, and we review the clinical, electrophysiological, and genetic data of previously reported patients with *ATP7A*-related isolated and complex dHMN.

## 2. Materials and Methods

Two brothers underwent clinical and electrophysiological evaluation. Laboratory testing, magnetic resonance imaging (MRI) or computed tomography (CT) scan, and genetic analysis were performed. Written informed consent for the use and publication of clinical and medical data was obtained from both individuals.

### 2.1. Patients

A 34-year-old male was examined at the Institute of Clinical Neurophysiology, University Medical Center Ljubljana, for the diagnosis of a neurological syndrome. Three years later, his older brother, at the age of 42 years, was referred to the Institute for neurophysiological evaluation due to gait difficulties.

### 2.2. Nerve Conduction Studies

Motor nerve conduction studies of the median, ulnar, tibial and fibular nerves were performed using disposable self-adhesive disk recording electrodes (019–400400, Natus Neurology Incorporated, Middleton, WI, USA) which were placed over the end-plate region of the abductor pollicis brevis, abductor digiti minimi, abductor hallucis and extensor digitorum brevis muscles, respectively. The standard bipolar felt pad stimulation electrode (019–401500, Nicolet Biomedical Incorporated, Madison, WI, USA) was positioned over the nerve trunk with the cathode 8 cm proximal to the motor point of the target muscle.

Sensory nerve conduction velocities and amplitudes of the sensory nerve action potentials of the median (second finger), ulnar (fifth finger) and sural nerves were studied by antidromic nerve stimulation. The same bipolar felt pad stimulation and detection electrodes were used. The cathode of the stimulating electrode and the active detecting electrode were 14 cm apart.

The nerve potentials were recorded by the EMG system (Nicolet, Natus Neurology Incorporated, Middleton, WI, USA).

### 2.3. Genetic Testing

We performed the extraction of genomic DNA samples from peripheral blood using Chemagic DNA Blood 4k Kit (Revvity, Waltham, MA, USA), and the quantity was determined with Qubit fluorimetric method. DNA was sheared with M220 ultrasonicator (Covaris, Woburn, MA, USA) and the exome enrichment was performed using the xGen^®^ Exome Research Panel v1.0 (IDT, Coralville, IA, USA). The exome library was sequenced using Illumina NextSeq 550 sequencer (Illumina, San Diego, CA, USA) in 150 base-paired end reads. Sequencing data were analyzed following GATK best practice guidelines. Short reads were aligned using BWA v0.7.2, and variant calling was performed with GATK v3.5. Variants were annotated and their potential impact predicted using snpEff v4.3, with additional pathogenicity scores obtained from the dbNSFP v2.0 database. Both classical (SIFT, PolyPhen-2, MutationTaster) and meta-predictors (MetaSVM, CADD, REVEL) were used to assess the functional effects of identified variants.

To determine the functional effect of the identified variant, we performed next-generation sequencing of the *ATP7A* messenger RNA transcript. After isolation of the RNA sample from peripheral blood of patient 1 and reverse transcription, we amplified exons 4– of the *ATP7A* transcript NM_000052.7, including the site affected by the identified splice variant. Subsequently, we performed deep sequencing of the amplified region and aligned the resulting sequences to the hg19 genome reference using the HISAT2 software and visually inspected the splicing defect in the IGV browser.

## 3. Results

### 3.1. Clinical Symptoms and Signs (Table 1)

#### 3.1.1. Patient 1

The proband presented with mild global developmental delay, noticed at 1–2 years of age. He walked independently by 18 months and spoke his first words after three years of age. In early childhood, he experienced regression, difficulties with fine motor coordination and speech articulation, including stuttering. Since infancy, he has had chronic diarrhea and, occasionally, urinary tract infections. In the course of the disease, he developed steppage gait. He attended a special needs school and completed eight years of education. Dysmorphic features included long face, high arched palate, coarse hair, mild kyphoscoliosis, and protruding shoulder blades.

**Table 1:** Clinical data in patients with *ATP7A*-related distal hereditary motor neuropathy.

	Patient 1	Patient 2	[14]	[12] Family 1/ Patient 1	[11]	[10] Family 1/ Patient 1	[10] Family 1/ Patient 2	[8] Family A with 9 pts	[8,15] Family B with 2 pts
*ATP7A* variant (NM_000052.7)	c.1544-2A>T		c.3544A>G; p.(Ile1182Val)	c.4236delA; p.(Lys1412AsnfsTer15)	c.2576A>G; p.(Asp859Gly)	c.2972C>A; p.(Ala991Asp)		c.4156C>T, p.(Pro1386Ser)	c.2981C>T, p.(Thr994Ile)
Functional studies	Yes, current study			Yes				Yes	Yes
Age of onset (years)	2	5	1.2	3	Childhood	27	Youth	4–61	2–8
First signs	Motor and speech delay	Chronic diarrhea, hyperhydrosis	Equinus gait	Gait difficulties, learning difficulties, orthostatic hypotension, temperature instability	Chronic diarrhea	Cramps, progressive difficulties in feet dorsiflexion	Chronic diarrhea		
Age at last examination (years)	34	42	42	24	42	47	43	16–62	26–29
Height (cm) (percentiles)	168 (P10)	181 (P50–P75)	N/A	N/A	164 (P5)	187 (P75–P90)	177 (P25–P50)	N/A	N/A
Weight (kg) (percentiles)	56.5 (P < 5)	83 (P50–P75)	N/A	N/A	51 (P < 5)	78 (P90–97)	69 (P50–75)	N/A	N/A
HC (cm) (percentiles)	57 (P25–P50)	59.5 (P75–P90)	N/A	N/A	56.5 (P50)	61.5 (P > 97)	62 (P > 97)	N/A	N/A
Peripheral nervous system	Motor neuropathy, proximal muscles weakness	Motor neuropathy, proximal muscles weakness	Proximal muscles weakness, distal motor neuropathy	Distal muscle weakness of legs	Distal muscle weakness and atrophy in LL	Distal axonal motor neuropathy in LL and UL	Distal axonal motor neuropathy in LL and UL	5/9 distal weakness and atrophy on LL and UL, 3/9 distal weakness on LL, 1/9 mild weakness on hands, 1/9 distal sensory loss on LL and UL, 4/9 distal sensory loss on LL	1/2 distal weakness and atrophy on LL and UL, 1/2 distal weakness on LL
Central nervous system	Cerebellar, extrapyramidal and pyramidal involvement, learning difficulties	Cerebellar involvement	No	Learning difficulties	Pyramidal involvement				
Autonomic dysfunction	Diarrhea, sinus bradycardia with individual supraventricular extrasystoles	Diarrhea, hyperhidrosis, paroxysmal atrial undulation—radiofrequency ablation, cardiovagal dysfunction	No	Orthostatic hypotension, temperature instability	Diarrhea, syncopes, bradycardia, orthostatic hypotension, cold-induced acrocyanosis and heat- induced acral erythema, erectile dysfunction	Diarrhea, hyperhidrosis, small fiber neuropathy, retrograde ejaculation with anejaculation	Diarrhea, hyperhidrosis, small fiber neuropathy, retrograde ejaculation with anejaculation		
Dysmorphic features	Coarse hair, long face, high arched palate	No	No	High forehead, large ears		Coarse hair, widows pick, sloping forehead, prominent eyebrows, anteverted nares, malar hypoplasia, micrognatia, long and thin face, long and stocky neck			
Skeletal, connective and cutaneous tissue abnormalities	Occipital horn, kyphoscoliosis, protruding shoulder blades	Occipital horn, cam-type impingement of femur’s neck	No	Occipital horn, pectus excavatum, hypermobile joints, lax skin	Occipital horn, joint laxity, thin skin	Occipital protuberance, anterior prominence of the hyoid bone, asymmetry of clavicles, limited elbow extension	Occipital protuberance, anterior prominence of the hyoid bone, asymmetry of clavicles, limited elbow extension, extensible and redundant skin		
Serum copper (normal range)	65.5 μg/dL (70–140)	54.2 μg/dL (70–140)	N/A	313 mg/L (620–1544)	11 μmol/L (>12.5)	47–63 μg/dL (70–140)	63–69 μg/dL (70–140)	12.5–15.4 nmol/L (11–23.6)	13.4–15.3 nmol/L (11–23.6)
Serum ceruloplasmin (normal range)	19 mg/dL (20–60)	18 mg/dL (20–60)	N/A	103 mg/L 170–460)	1.61 μmol/L (2)	14.7–19.1 mg/dL (20–60)	19.8–20 mg/dL (20–60)	N/A	N/A
EMG	Axonal motor neuropathy	Axonal motor neuropathy	Axonal motor neuropathy	Axonal motor neuropathy	Axonal motor neuropathy	Axonal motor neuropathy	Axonal motor neuropathy	7/9 Axonal motor neuropathy	1/9 Axonal motor neuropathy
Brain MRI/CT	Occipital horn, cerebral and cerebellar atrophy	Occipital horn, mild frontal cerebral atrophy	Normal	Elongated and tortuous anterior and middle cerebral arteries					
EEG	Intermittent theta	Normal							
Others	Chronic urinary tract infections	Myopia	Malignant hyperthermia during surgery	dHMN worsened while treated with copper replacement therapy	Pollakisuria, sudden death at the age of 46 ys				

dHMN—distal hereditary motor neuropathy, HC—head circumference, LL—lower limbs, UL—upper limbs.

Neurological evaluation during childhood revealed classical signs of motor neuropathy, including bilateral foot drop, symmetrical distal wasting and weakness in the upper and lower limbs with Achilles areflexia. At age of 22 years, additional neurological findings were noted, including proximal muscle weakness and signs of cerebellar, extrapyramidal and pyramidal system involvement (temporal and masseter muscle weakness, knee flexion and extension weakness, saccade eye movements, mild hypomimia, torticollis, axial and upper limb rigidity, exaggerated superficial and deep abdominal and patellar reflexes). His disease slowly progressed, with the development of balance difficulties and a wider gait in recent years. At his last evaluation, at the age of 34 years, he was living with his mother and working daily in a regional occupational activity center. Mild intention tremor and ataxic gait were additionally noticed. He had no significant joint contractures, joint hyperlaxity or palpable occipital horns.

**Figure 1:**
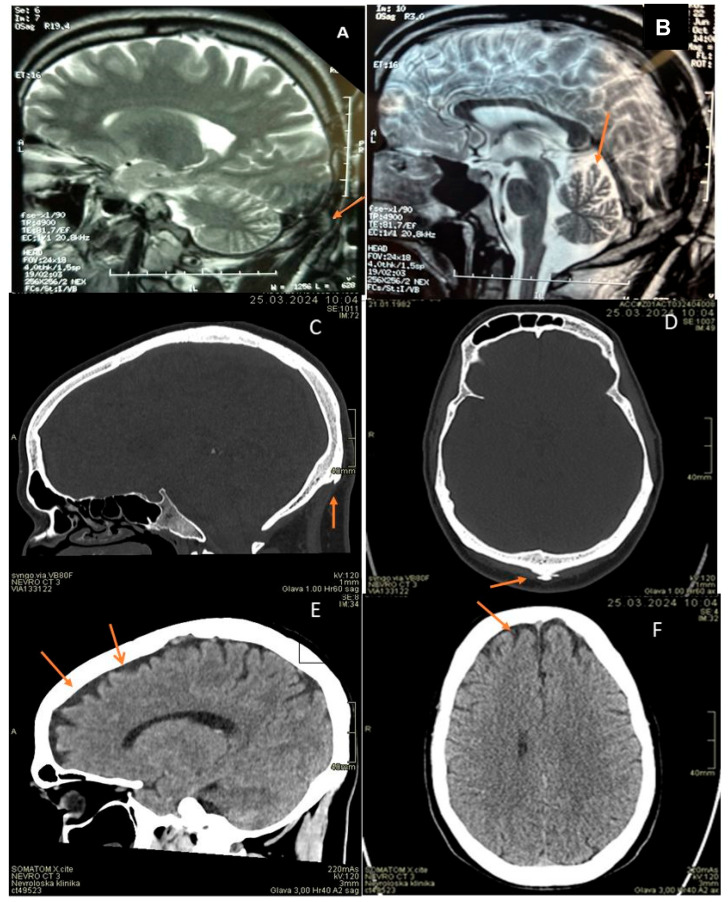
(**A**): patient 1, brain MRI, T2 sagital image: occipital horn (orange arrow). (**B**): patient 1, brain MRI, T2 sagittal image: dilated frontoparietal and cerebellar sulci (orange arrow). (**C**,**D**): patient 2, head CT, bone window, sagital and axial planes: occipital horn (orange arrows). (**E**,**F**): patient 2, head CT, soft tissue window, sagittal and axial planes. Mildly dilated frontal sulci (orange arrows).

Investigations revealed EMG consistent with axonal motor neuropathy (Table 2). In EEG, posterior background activity was slower due to intermittent theta (6 Hz) frequency. His head MRI showed occipital horn and mild cerebral and more pronounced cerebellar atrophy (Figure 1). Additionally, sinus bradycardia with individual supraventricular extrasystoles was noticed on ECG and spirometry showed moderate obstructive-restrictive ventilatory abnormalities. His serum copper and ceruloplasmin levels were slightly reduced (Table 1).

#### 3.1.2. Patient 2

The proband’s elder brother presented with frequent diarrhea and hyperhydrosis, first noted at the age of 5 years. In high school, at the age of 16 years, he experienced difficulties with long-distance running. Since the age of 27 years, he has had occasional muscle cramps in his legs. At the age of 36 years, he noticed hand tremors and clumsiness in the hands. At the age of 39 years, he was diagnosed with atrial fibrillation and started on anticoagulant therapy and a beta-blocker. Two years later, he underwent radiofrequency ablation. Since the age of 40 years, his gait problems have progressed significantly. At the time of examination, at the age of 42 years, he was no longer able to stand on his toes or heels, nor could he run. He graduated from a secondary school of economics and works as store cashier.

Neurological examination revealed slight muscle wasting and weakness (MRC 4/5) in distal parts of upper limbs with normal myotatic reflexes. On lower limbs, he had muscle wasting and weakness distally from the knees with Achilles areflexia. He was unable to stand up from a squat without support, was not able to stand on toes or heels and had a peroneal gait. Additionally, he had slight gaze dysmetria, intension tremor, and lower limb ataxia. He had no sensory loss, no pyramidal or extrapyramidal abnormalities, no micturition or sexual dysfunction, no cognitive decline, and no dysmorphic features.

Nerve conduction studies showed signs of axonal motor neuropathy (Figure 1). EEG was normal. His head CT scan showed occipital horn and mild frontal cerebral atrophy (Figure 1). An electrocardiogram performed after radiofrequency ablation showed sinus rhythm. Spirometry showed mild obstructive ventilatory abnormalities. He had myopia (−6 OD and −6.5 OS). His serum copper and ceruloplasmin levels were slightly reduced (Table 1).

#### 3.1.3. Family History

Family history was unremarkable. The patients had no children and no maternal uncles, and all six maternal male cousins were healthy (Supplement Figure S1). The mother did not report any health issues and declined genetic testing.

### 3.2. Genetic Testing

Exome sequencing in the patients identified a novel hemizygous splice-site variant (NM_000052.7:c.1544-2A>T) in *ATP7A*. Several lines of evidence support the pathogenic nature of this variant, including in silico algorithms for prediction of splicing effect (combined prediction of MaxEntScan and PWM algorithms in scSNV database was 0.99, which indicates a high likelihood of a splice defect, and Human Splice Finder 3.1 predicted that the variant most probably affects splicing). Furthermore, there is a report of another splice-site variant affecting the neighboring nucleotide in the same acceptor site, which was classified as (likely) pathogenic in association with *ATP7A*-related phenotypes (c.1544-1G>A, ClinVarID:210394). The variant is also absent in worldwide populations (gnomAD).

**Figure 2:**
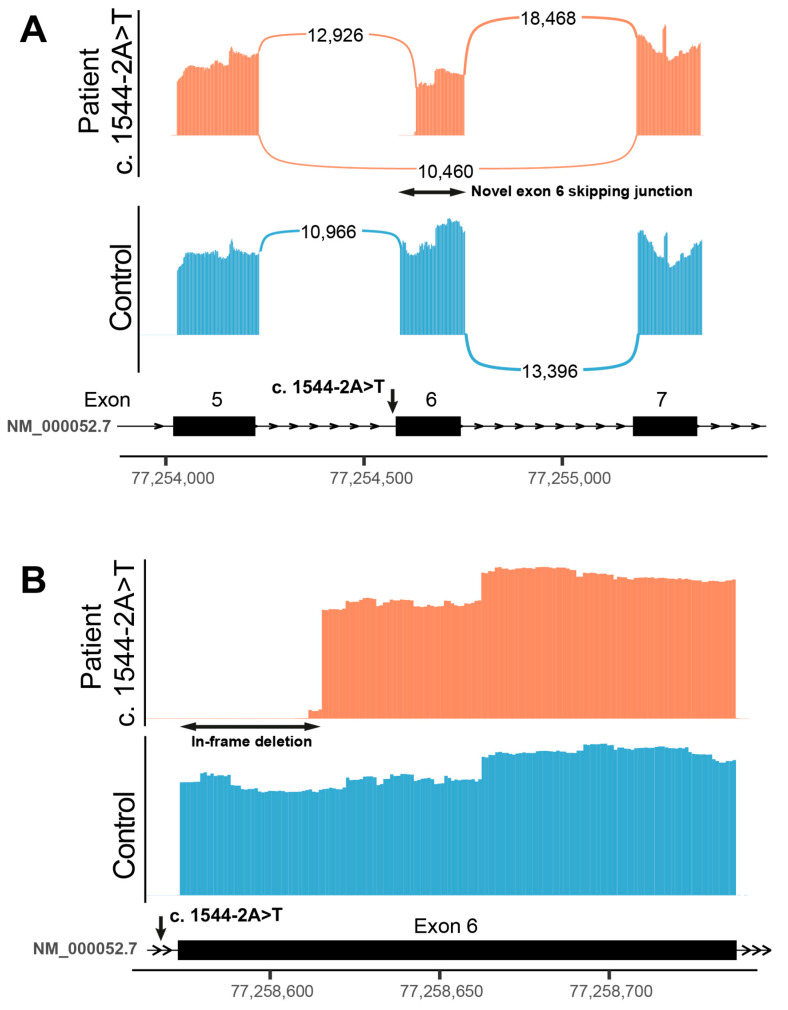
Results of the RNA splice analysis. Panel (**A**) shows the Sashimi plots of exon junctions, adjacent to the splice variant site. The profile of splice junctions in patient 1 (marked in red) versus a normal control (marked in blue) indicates creation of a novel splice junction between exons 5 and 7 in a proportion of reads, which suggests skipping of exon 6. The panel (**B**) shows a zoomed coverage profile of exon 6 in the patient (marked in red) versus a normal control (marked in blue). The 5′ prime region of exon 6 is deleted in all transcripts where this exon has not been skipped. This deletion is predicted to result in an in-frame deletion of 14 amino acids in the ATP7A protein.

The results of RNA analysis indicated that the splice variant results in creation of two major aberrant transcript isoforms: (1) a transcript isoform with skipping of exon 6, which is predicted to result in the loss of the reading frame and likely leading to the non-sense mediated decay of this isoform, and (2) a transcript isoform with 5′ truncation of 42 nucleotides from the exon 6 sequence, which is predicted to result in an in-frame deletion of 14 amino acid residues in the final protein product (Figure 2). Based on the coverage of detected aberrant junctions, we estimate that the exon skipping and in-frame deletion isoforms were present in 44.7% and 55.3% of transcripts, respectively. No presence of residual physiological isoform was detected. Considering that the deletion of 14 amino acid residues in the latter isoform does not affect a functionally significant domain of the ATP7A protein, it is possible that this isoform preserves some residual functionality.

Using the available evidence favoring pathogenicity of the variant and the results of our functional assessment, we classify this variant as pathogenic according to the American College of Medical Genetics and Genomics (ACMG) guideline (PVS1, PS3, PM2, and PP4) [16]. No other likely pathogenic or pathogenic variants were observed in the exome sequencing data.

## 4. Discussion

Among the phenotypes associated with pathogenic *ATP7A* variants, MD and OHS are the most frequently reported, while *ATP7A*-related distal hereditary motor neuropathy (dHMN) has only been reported in six families to date [8,10,11,12,14,15].

dHMN was initially recognized as a distinctive clinical presentation in association with two missense *ATP7A* variants (c.4156C>T, p.P1386S and c.2981C>T, p.T994I) in males from two families who presented with late, often adult-onset distal motor neuropathy showing no other clinical features and who had normal levels of serum copper [8,15]. As seen in some family members from these original pedigrees—and later supported by further reports—dHMN may begin as early as childhood [8,14,15]. However, progression is usually slow, and diagnosis is often delayed until adulthood, as in our two patients. The condition frequently presents with foot deformities and is often accompanied by absent or diminished Achilles tendon reflexes, with upper limb involvement developing in later stages. The electrophysiological findings, summarized in Table 2, showed reduced compound motor action potentials, normal or only mildly decreased motor conduction velocities, and preserved sensory nerve conduction parameters. Serum copper levels were mildly reduced in most patients [10,11,12,14], though within the normal range in some [8], while serum ceruloplasmin level was generally slightly below the lower limit of normal [10,11,12,14].

Although MD, OHS and dHMN are considered distinct phenotypes within the spectrum of *ATP7A*-related copper transport disorders, phenotypic overlap between MD and OHS has long been recognized [17]. Only recently, however, has overlap between OHS and dHMN been described [11]. To date, four individuals with combined features of OHS and dHMN have been reported in the literature [10,11,12], and both of our patients also exhibited this phenotype. In the majority of patients (5 of 6), clinical symptoms were noted in early childhood, with chronic diarrhea reported as the first symptom in half of them [8,9] and current study. As the condition progressed, additional signs of autonomic nervous system dysfunction emerged (e.g., temperature instability, orthostatic hypotension, syncope, retrograde ejaculation), followed by symptoms consistent with dHMN. An occipital horn was confirmed by imaging in four reported patients, while an occipital protuberance was described in two others. One of the patients reported here also exhibited mild global developmental delay in early childhood and central neurodegeneration, which, with age, led to clinical signs of cerebellar, extrapyramidal and pyramidal system involvement and mild intellectual disability—clinical manifestations not previously reported in patients with *ATP7A*-related dHMN. All patients had in common that these signs were generally unrecognized as part of an *ATP7A*-related disorder, with diagnosis typically delayed by nearly 30 years after symptom onset.

Although a clear genotype–phenotype correlation has not been established, truncating variants (nonsense or frameshift variants, as well as large deletions or insertions) correlating with an absence of functional protein are largely associated with the severe phenotype of classic MD, while variants that permits some residual ATP7A protein activity (including some splice-site, intronic, and missense variants) result in atypical MD or OHS [7,13]. In the first report of patients with dHMN, two different missense *ATP7A* variants in transmembrane domains were described (Table 1; [6]), inducing abnormal ATP7A intracellular trafficking and resulting in excessive ATP7A localization at the plasma membrane [9]. Subsequently, a patient with an overlapping OHS and dHMN phenotype and a C-terminal truncating *ATP7A* variant was reported (Table 1; [12]). Late truncating *ATP7A* variants, leading to a truncated ATP7A predominantly localized at the plasma membrane, have previously already been associated with OHS phenotype, but dHMN phenotype has not been reported in those patients [18,19]. While most reported *ATP7A*-related dHMN cases have involved missense variants (five of six families) and only one late-truncating variant, the brothers in this report were found to have a novel splice-site variant (NM_000052.7:c.1544-2A>T), thereby expanding the known mutational spectrum associated with the overlapping OHS/dHMN phenotype. An RNA analysis in the patient revealed the presence of two major aberrant transcript isoforms: one isoform likely leads to the non-sense mediated decay, and the second isoform might lead to a protein product with some residual functionality preserved as it does not lead to premature termination and consequent nonsense-mediated decay. Interestingly, a splice-site variant affecting the adjacent nucleotide (c.1544-1G>A) has previously been reported in the ClinVar database (ID: 210394) in two individuals with *ATP7A*-related phenotypes—one diagnosed with MD and the other with dHMN. In our two siblings, the severity of the dHMN phenotype was comparable; however, their neurodevelopmental outcomes differed. The younger brother exhibited developmental delay in early childhood and learning difficulties, whereas the older brother maintained normal cognitive function. Inter- and intrafamilial variability has been previously documented in *ATP7A*-related disorders [20]. As recently reviewed by Harkness et al. (2024) [21], phenotypic variability among individuals with *ATP7A* splice-site variants likely results from multiple contributing mechanisms, including the inherent leakiness of splicing and factors influencing the regulation of ATP7A expression.

## 5. Conclusions

The clinical presentation of individuals with overlapping OHS/dHMN phenotype can be nonspecific in the early stages, often resulting in diagnostic delays of several decades. Dysautonomia is frequently the first and most prominent symptom, with signs of peripheral neuropathy typically emerging later in the disease course. In cases of recurrent unexplained childhood diarrhea, clinicians should consider *ATP7A*-related disorders in the differential diagnosis and proceed with further investigations—such as radiological imaging to identify pathognomonic occipital horns, electrophysiological studies to detect peripheral neuropathy, and genetic testing for molecular confirmation. The mutational spectrum associated with the OHS/dHMN phenotype is broader than previously recognized and includes not only missense and late truncating variants but also *ATP7A* splice-site variants. As illustrated by the present family and others, detailed clinical descriptions of individuals with novel *ATP7A* variants contribute to a better understanding of genotype–phenotype correlations within this broad disease spectrum.

## Figures and Tables

**Table 2:** Electrophysiological data in patients with *ATP7A*-related distal hereditary motor neuropathy.

	n. Median	n. Ulnar	n. Fibular	n. Tibial	n. Median	n. Ulnar	n. Sural
	CMAP (mV), MNCV (m/s)	CMAP (mV), MNCV (m/s)	CMAP (mV), MNCV (m/s)	CMAP (mV), MNCV (m/s)	SNAP (µV), SNCV (m/s)	SNAP (µV), SNCV (m/s)	SNAP (µV), SNCV (m/s)
Patient 1	**3.8, 50.0**	**3.1**, 56.0	**0.2**, 40.0	5.4, **40.0**	56.0, 64.0	41.0, 61.0	14.0, 54.0
Patient 2	11.4, 58.0	9.6, 60.0	**0.7, 38.0**	5.6, **41.0**	27.0, 57.0	59.0, 53.0	10.0, 48.0
[14]	8.0, 56.0		**1.6**, 42.0 (R) **2.5**, 46.0 (L)	**4.8**, 46.1 (R) **5.0**, 42.9 (L)			
[12] Patient 1			**0.6**, 47.3				9.1, 56.7
[12] Patient 2			2.6; 55,4				
[11]	**6.2**, 57.3 (R) 10.4, 52.9 (L)	15.2, 59.2	**1.2, 39,7** (R) **2.6**, 44.7 (L)	**3.2**, 40.9 (R) **3.8, 39.4** (L)	16.5, **35.8** (R) 20.7, **43.1** (L)	15.3 (R) 15.5, 45.3 (L)	22.6, **32.8** (R) 20.6, **32.1** (L)
[10] Patient 1	7.3		**0.1,** 45.9	**1.1**			**5.4**
[10] Patient 2	15.0		**0.2, 34.1**	**0.8**			10.9
[8] Family A with 9 pts:							
A/IV-1	4.3, 57.6			**1.9**			**13.3**
A/IV-2	**2.62**, 61.5			**2.80**			26.6
A/IV-3	**3.6**, **46.8**						**17.0**
A/IV-6				**2.73**			
A/IV-7	12.0, 57.5			**2.3**			
A/IV-12	**0.03**			**0**			
A/V-1	6.3, 64.7			13.5			
A/V-2	**2**						
A/V-3	8.0, 50.5			4.8			
[8] family B with 2 pts:							
B/IV-2	**2.7**, 50.5						25.8
B/IV-3							62.0

CMAP—compound motor action potential, L—left, MNCV—motor nerve conduction velocity, R—right, SNAP—sensory nerve action potential, SNCV—sensory nerve conduction velocity, **bold**—lower than 2 SD of normal values.

## Data Availability

The original contributions presented in this study are included in the article. Further inquiries can be directed to the corresponding author.

## Supplementary Materials

The following supporting information can be downloaded at: https://www.mdpi.com/article/10.3390/genes16091077/s1, Figure S1: Pedigree of the family with two affected brothers.
