# Supplementary figures and images for: Novel ATP7A Splice-Site Variant Causing Distal Motor Neuropathy and Occipital Horn Syndrome: Two Siblings and Literature Review
Source: Genes (Basel). 2025 Sep 15;16(9):1077. doi: 10.3390/genes16091077 (PMC12469850; doi:10.3390/genes16091077)

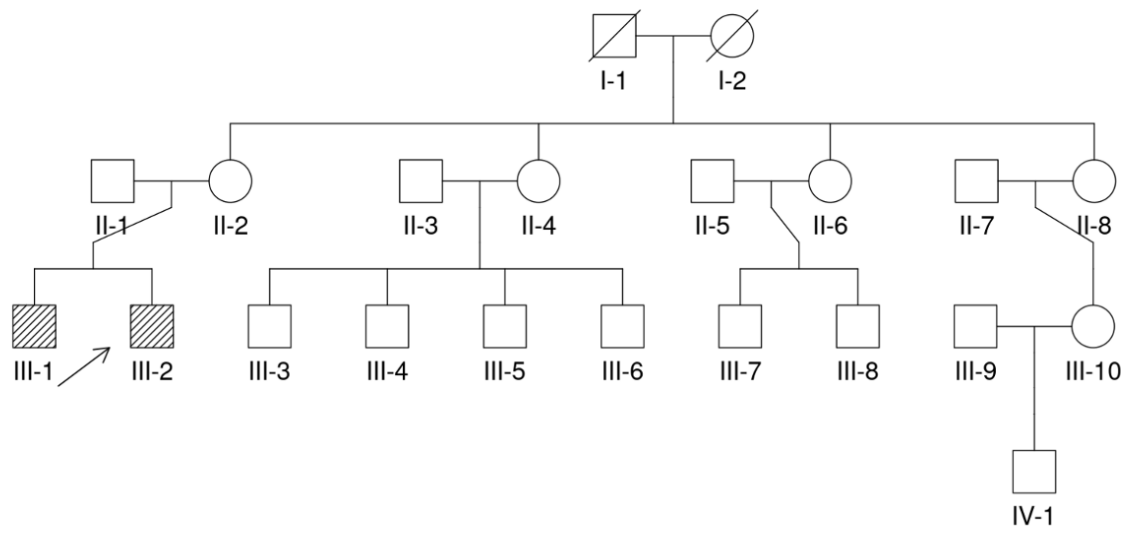

Figure 1, Supplement: Pedigree of the family with two affected brothers

Supplement: Supplementary file 1 [file genes-16-01077-s001.zip › genes-3820436-supplementary.pdf]
